# Embolism of the Axillary Artery

**Published:** 1884-04

**Authors:** Addison H. Foster

**Affiliations:** Chicago


### ARTICLE VI.

EMBOLISM OF THE AXILLARY ARTERY. By ADDISON H. FOSTER, M.D., Chicago.

The case to which attention is here directed, was one occurring in the practice of Dr. Wm. E. Clarke, of this city, through whose courtesy I was several times enabled to make observations of it. I submitted a report of the same to the Chicago Medical Society in the year 1875, from rather full notes taken at the date of observation, which have unfortunately been since mislaid. The following report is based only upon some memoranda taken at that date and recently found, as also upon the points which were at the time of observation more especially impressed upon my memory.

The subject of the attack was a female, about 35 years old, married, and the mother of several children. She was of rugged family and had enjoyed sound health until specifically infected by her husband; after which she was continually ill and suffered severely with metritis and anteflexion of the uterus. She was particularly liable to precordial distress and irregular action and palpitation of the heart, with accompanying hysterical phenomena.

About a year previous to the occurrence of the embolism a roughness became audible with the first sound of the heart, but with no apparent organic cardiac change.

On the 16th of January, 1874, she was suddenly seized with an agonizing pain in her left arm; most intense in the axilla, at the bend of the elbow, and along the wrist and hand. She complained of cold in the arm, but of no general chilliness, of numbness and defective sensations and inability to move the part.

The surface was cold to the touch and purplish in color. Upon examination no radial, ulnar, nor brachial pulsation could be detected. The heart's rhythm was irregular, with a decided systolic bruit, and the pulse about 90 to the minute. The patient was kept quiet in bed; the arm wrapped in cotton batting, and artificial heat applied in order to maintain a normal temperature. After about three weeks the radial pulse began to return very feebly; it slowly grew stronger, but never attained its former strength and fullness. The pain contained to be severe for about six days and then gradually subsided, with occasional accessions quite sharp but brief in duration.

The surface temperature remained low for months. Desquamation followed in July. The arm remained permanently weakened and of small size.

The patient continued to live in variable, but yet generally poor health, and with very intractable uterine disease. There were occasional cardiac disturbances and consequent apprehension of impending danger to the limb or to life. There were also hepatic derangements of more or less constant occurrence.

On the 29th of October, 1874, a species of broncho-pleuro-pneumonia invaded the right lung, and in ten days was accompanied with a copious expectoration of offensive pus. The dyspnœa, palpitation, and weakness increased until death occurred on the 15th of November. Unfortunately, the pathological anatomy of the case was not cleared up by a post-mortem examination.

In reviewing its feature it is evident that the embolus was mechanical rather than septic in its nature, and was derived probably from the valvular margins of the heart, as the occluding plug on the arterial side of the general circulation is generally from that source or from clots among the columnæ carneæ.

Accounts of embolism in the upper extremities with recovery, are not numerous. The recoverymay take place, as in other parts, by the collateral circulation being promptly established, and by the disintegration or resolution of the embolus.

Literature throws meager light upon the theory that specific poison is the cause of the cardiac lesions, although it may be admitted as possible.

That the migratory plug chanced to lodge in the axillary artery rather than in the left middle cerebral—the more frequent occurrence—naturally prolonged the history of this case. although it is claimed that even in the latter event recovery sometimes results.

An arterial embolus from the left side of the heart without septic properties would rarely account for an abscess of the lung, as a benign subpleural infarction of the lung would not be expected to break down into a large quantity of pus.

A venous embolus of septic or putrid origin would not be anticipated from any known condition of the patient.

It could be more readily imagined that the pus had an hepatic origin, a supposition perhaps not incompatible with the state of the case.

